# Jean-Martin Charcot: Pioneer of Neurology

**DOI:** 10.7759/cureus.66762

**Published:** 2024-08-13

**Authors:** Renish N Contractor, Manav Shah, William Manwell, Kalani J Dempsey, Prathap Simhadri

**Affiliations:** 1 Urology, Florida State University College of Medicine, Daytona Beach, USA; 2 Biomedical Sciences, University of Florida, Gainesville, USA; 3 Internal Medicine, Lake Erie College of Osteopathic Medicine, Bradenton, USA; 4 Biomedical Sciences, University of West Florida, Pensacola, USA; 5 Internal Medicine/Nephrology, AdventHealth, Florida State University College of Medicine, Daytona Beach, USA

**Keywords:** historical vignette, jean-martin charcot, neurology, charcot's disease, medical history

## Abstract

Jean-Martin Charcot, born on November 29, 1825, in Paris, France, is known as the father of neurology. During a time when neurology was not yet a recognized medical specialty, Charcot's pioneering contributions significantly advanced the field. Charcot's use of the anatomo-clinical method, which correlates clinical symptoms with anatomical findings, led to the discovery and characterization of numerous neurological conditions, including multiple sclerosis (MS), amyotrophic lateral sclerosis (ALS), Charcot’s joint, and Charcot-Marie-Tooth (CMT) disease. His methodical approach to documenting clinical signs and conducting post-mortem examinations revolutionized neurological research and diagnosis, laying the groundwork for modern neurology. The anatomo-clinical methods continue to be a vital tool in neurological research and practice today. Charcot's work extended beyond clinical practice, influencing the study of neurology through his role as an educator and mentor to many, including Sigmund Freud. Despite some controversies and a reputation for being difficult to work with, Charcot's legacy endures, with his initial discoveries fostering greater awareness and the development of therapies for various neurological disorders.

## Introduction and background

Early life and education

Jean-Martin Charcot, known as the father of neurology, was born in Paris, France, on November 29, 1825 [1]. Charcot was born at a time when neurology was not a recognized field in medicine [2]. Charcot was one of four siblings from a lower-income family. Due to the financial restrictions of Charcot’s family, their father decided only the one with the highest scores would proceed for further schooling [3]. Charcot excelled against his siblings and was able to begin his career in medicine. Charcot matriculated into the University of Paris for medical training in 1848 [2]. Charcot graduated in 1853 and presented his thesis on the difference between the symptoms and lesions of gout from chronic rheumatism, which at the time were considered the same [3]. Charcot’s method of presentation, through theatrical demonstration and live interviews, helped him stand out from other prominent individuals of the time [2]. Following graduation from medical school, Charcot started his career by taking an internship at L’Hôpital Salpêtrière in Paris [2]. Alongside Charcot’s academic career, he married a wealthy widow, Madame Durris, in 1962 and had two kids, Jeanne in 1865 and Jean-Baptiste in 1867 [1]. Charcot’s son Jean-Baptiste followed in his father's footsteps as a doctor of medicine even though he had a passion for being a maritime explorer [4].

## Review

Early career

**Figure 1:**
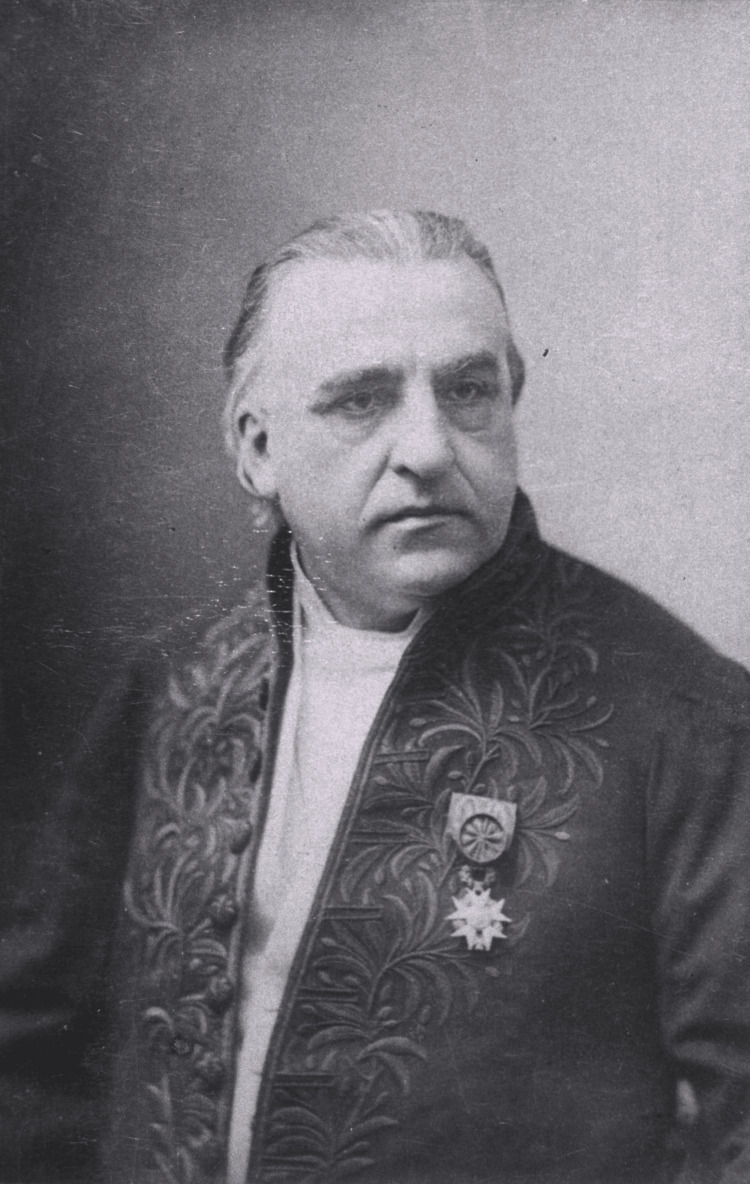
Portrait of Jean-Martin Charcot Credit: US National Library of Medicine [8]. This image is distributed under the terms of the Creative Commons Attribution-NonCommercial 4.0 International License.

Charcot (Figure 1) made many discoveries throughout his career, including the discovery of multiple sclerosis (MS) and Parkinson’s disease [4]. Charcot began his career as an intern at the L’Hôpital Salpêtrière in Paris [2]. Charcot’s thesis on the difference between gout and chronic rheumatism enabled him to receive the title “Chef de Clinique” in 1853 [2]. Chef de Clinique is the term given to those residents in medical school who have completed their specialist training in France [5]. Charcot held this position for three years before shifting paths to “physician to the hospital of Paris” in 1856 [6]. In 1860, he became a professor at the University of Paris, and he began a lifelong association with the Salpêtrière Hospital in Paris in 1862. He remained at the University of Paris until 1893. In 1882, he opened what was to become the greatest neurological clinic of the time in Europe. Charcot attracted students from all over the world, including Sigmund Freud, in 1885. Charcot's interest in hypnosis is what subsequently led to Freud's interest in the psychological origins of neurosis [7].

Hysteria

Charcot's work on hysteria shaped the fields of neurology and psychiatry at the time. At the Salpêtrière Hospital in Paris, Charcot noted the symptoms of hysteria. He identified a range of motor and sensory disturbances, seizures, and emotional outbursts. He termed the concept of "hystero-epilepsy," where he distinguished hysterical seizures from epileptic fits and utilized hypnosis both as a diagnostic tool and therapeutic method. Charcot's use of photography and illustrations to document these symptoms was groundbreaking at the time. This helped to educate other healthcare professionals and the public about hysteria [2]. He thought that hysteria was neurologically based, which was a shift from the previous school of thought of being purely psychological. This theory influenced his student, Sigmund Freud, and helped lay the foundation for psychoanalysis [7]. Despite facing criticism from his colleagues, Charcot's methods and diagnostic techniques helped our current understanding of hysteria, establishing it as a medical condition [7].

Anatomo-clinical method of neurology

Charcot’s research early in his academic career, between 1862 and 1875, was rooted in the anatomo-clinical method, a two-part method. This method linked clinical signs and symptoms with anatomical lesions [9]. The first step of the method included documenting clinical signs over a period of time. The second step occurred when the patient died and involved an autopsy of the patient’s brain and spinal cord. Combining both clinical signs and anatomical data from the autopsy, Charcot proposed clinical-anatomical correlations. Using the anatomo-clinical method, Charcot helped to define the tracts and nuclei of the neurological tract and defined findings as normal or abnormal. This helped link neurological disease to anatomical lesions. The anatomo-clinical method led to many discoveries and is Charcot’s most important contribution to clinical neurology.

This method led to Charcot’s discovery of motor degenerative diseases, such as amyotrophic lateral sclerosis (ALS), also known as Charcot disease. Charcot’s initial work with this method led to the discoveries of linking strokes to cortical localization theory and differentiating between Parkinson’s disease and MS [10]. Cortical localization theory led neurologists to understand that specific brain regions controlled specific motor, sensory, and language functions. His work helped define specific clinical signs of localized lesions in MS.

Although Charcot’s work using the anatomo-clinical method led to discoveries within the field of neurology, some of his studies and conclusions led to disapproval among his peers. Additionally, Charcot had a reputation for being difficult to work with, authoritarian, and intolerant of differing viewpoints. These incidents stained his reputation at the end of his career. Most of Charcot’s heritage has not survived well as the Charcot Museum disappeared, and the Bibliotheque Charcot carries very few of his manuscripts and collections [11].

Contribution to MS

In 1868, using his anatomo-clinical method, Charcot described “sclérose en plaques disséminées” (MS) [12]. MS had been described as early as the 14th century, but it was Charcot who correlated the postmortem pathologic findings and the clinical features of MS [9]. One important patient that helped Charcot with his findings was his servant named Luc. Luc had progressive tremors in her head and extremities that were exacerbated by movement and relieved by rest. When she died in 1866, Charcot performed an autopsy and observed numerous plaques in the brain and spinal cord, which were almost identical to each other. Charcot then went on to diagnose Luc postmortem with the cerebrospinal form of MS [13]. He would later describe the various forms, including cephalic, spinal, and mixed/cerebrospinal. He was also the first person to diagnose a living patient with MS. To help do this, he created “Charcot’s triad,” which included nystagmus, intention tremor, and scanning speech. This triad was not specific, but it was important as it attempted to separate MS from similar pathologies [14]. This discovery led to the understanding and distinction between MS and Parkinson's disease.

ALS

**Figure 2:**
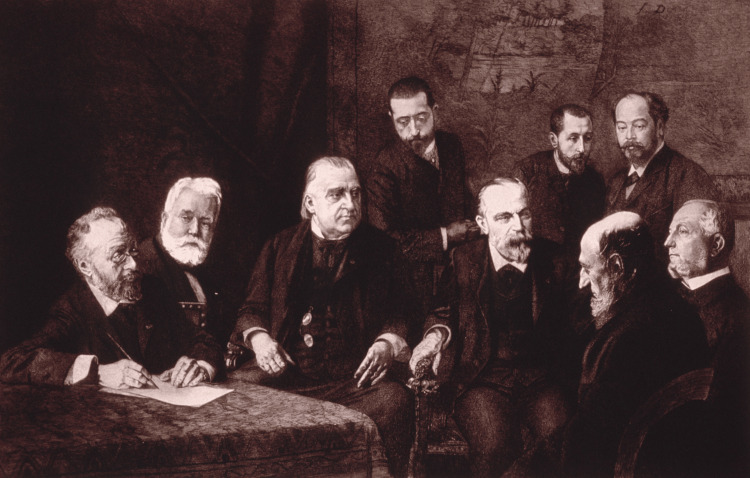
Jean-Martin Charcot consulting with his fellow colleagues Credit: US National Library of Medicine [19]. This image is distributed under the terms of the Creative Commons Attribution-NonCommercial 4.0 International License.

Charcot’s disease, more commonly known as ALS, is one of Charcot’s most famous neurologic discoveries [15]. Charcot served as the physician in charge of a chronic care hospital, where he focused his studies on cases of chronic and progressive weakness (Figure 2). He conducted these studies using the anatomo-clinical method between 1865 and 1869 when he first noticed a case of a young woman with progressive weakness and increased muscle tone with no sensory abnormalities and intact urinary function [16]. He found bilateral and symmetric sclerosis of the lateral column and acute amyotrophy of the anterior horn of the spinal cord. Lesions of the lateral column led to progressive paralysis and contractures, while lesions of the anterior horn led to paralysis without contractures [10]. In the Salpetriere, Charcot followed his patients very closely, observing their progressive functional decline with documentation recording the events. His discovery supported his theory that the location of the lesion led to various symptoms and that the motor component consisted of two parts [17]. Charcot’s findings became a cornerstone of modern-day neurology. Due to Charcot’s contributions, we now understand that gray matter motor nuclei damage results in weakness with associated muscular atrophy, while white lateral column damage leads to weakness with contractures and spasticity [18].

Charcot’s joints

Charcot was the first to describe joint pain in tabes dorsalis. He hypothesized that “…the arthropathy of ataxic patients seems to always start after the sclerotic changes have taken place in the spinal cord.” After the acute damage and inflammatory process, there is a healing process, which leads to a joint deformity. While syphilis was the most common cause in the 19th century, diabetes mellitus was found to have a correlation with Charcot’s joint, as recognized by William Jordan [14]. Up to 35% of diabetics develop Charcot’s joints, with most of them having diabetes for at least 10 years [3]. Charcot’s joint most commonly affects the feet and increases the risk of osteomyelitis and ulcers [10]. The pathomechanics are unclear at this time [18].

Charcot-Marie-Tooth (CMT) disease

CMT disease was first described in 1884 by Charcot, Pierre Marie, who was his resident, and Howard Henry Tooth [15]. They described five cases of “progressive muscular atrophy, often familial,” eventually also known as peroneal muscular atrophy. In these cases, they were the first to note that the disease was a neuropathy rather than a myopathy. CMT has come to become the most commonly inherited neuromuscular disorder, with 25 associated genes [20]. These genes often affect myelin, Schwann cells, and axons [21]. CMT is characterized by slowly progressive foot deformities, known as pes cavus, sensory loss, weakness found in the lower extremities, and reduced deep tendon reflexes. Symptoms start in adolescence or the teenage years, with weakness starting in the lower extremities and later progressing to the upper extremities.

Visual art in the neurologic career of Jean-Martin Charcot

Jean-Martin Charcot incorporated visual art into his normal day-to-day practice of neurology [10]. His innate artistic talents were due to his visual perception and remarkable memory. His drawings first started as a hobby and then later became scientific. His drawings correlated to his anatomo-clinical method and portrayed his patients’ postures and clinical neurological signs. Some of these are showcased in the book “Charcot, une vie avec l'image,” published by Catherine Bouchara in 2013 [14]. Charcot used historical artistic representations from past centuries to help further his work regarding the development of hysteria. He did his most expressive work when under the influence of cannabis [18]. Although there were benefits to his art and imagery use, art also misguided Charcot's career. One specific example is when he attempted to convince critics that certain disorders seen at the Salpêtrière Hospital in Paris, France, were independent of his suggestive influence. Overall, Charcot was essential in incorporating the usage of medical photography into the field of neurology [14].

Later life

Charcot lived an almost sedentary life in that he did little to no physical activity alongside indulging in health-impacting activities such as smoking an excessive number of cigars [1]. In 1893, on his wife's insistence, Charcot and two old pupils, Professor Debore and Straus, went on vacation to Morvan, but Charcot suffered from myocardial infarction and pulmonary edema on vacation in August [4]. In 1893, he died at the age of 68, leaving a legacy that has shaped modern neurology. Charcot’s funeral service took place in the chapel of his alma mater, the Salpêtrière [2]. Charcot was buried at the Montmartre Cemetery, and in 1895, a bronze statue was erected outside of the Salpêtrière in Paris; the statue was paid for by pupils throughout his life. Unfortunately, his statue was used as furnace fuel during German occupation during the Second World War [4].

## Conclusions

Jean-Martin Charcot, born on November 29, 1825, in Paris, France, left a mark on the field of neurology, earning him the title of the father of neurology. At a time when neurology was not yet recognized as a distinct medical specialty, Charcot's efforts were critical in its development and acceptance. His use of the anatomo-clinical method, which linked clinical symptoms with anatomical findings, led to major breakthroughs in the understanding and diagnosis of neurological conditions such as MS, ALS, Charcot’s joint, and CMT disease.

Beyond his clinical work, Charcot's served as an educator and mentor to many, including Sigmund Freud, further cemented his impact on medicine. Despite facing controversies and a reputation for being challenging to work with, he was dedicated to advancing medicine, demonstrating his commitment to improving patient care and understanding of neurological disorders. Charcot's legacy endures in the increased awareness and development of therapies for various neurological conditions, solidifying his position as a foundational figure in the field of neurology. Charcot’s initial discoveries transformed neurological research and practice and laid the groundwork for future innovations.
